# 405. Incidence and Potential Risk Factors of COVID-19 Associated Pulmonary Aspergillosis (CAPA)

**DOI:** 10.1093/ofid/ofad500.475

**Published:** 2023-11-27

**Authors:** Waki Imoto, Koichi Yamada, Yukihiro Kaneko, Hiroshi Kakeya

**Affiliations:** Osaka Metropolitan University Graduate School of Medicine, Osaka, Osaka, Japan; Osaka Metropolitan University Graduate School of Medicine, Osaka, Osaka, Japan; Osaka Metropolitan University Graduate School of Medicine, Osaka, Osaka, Japan; Osaka Metropolitan University, Osaka, Osaka, Japan

## Abstract

**Background:**

Invasive pulmonary aspergillosis (IPA) that develops in coronavirus disease 2019 (COVID-19) patients is called COVID-19 Associated Pulmonary Aspergillosis, CAPA. The incidence of CAPA is reported as high as 1–40 % worldwide. However, the incidence of CAPA is highly variable among reports and it may vary among countries and medical facilities. Therefore, we investigated the incidence of CAPA in severe to critical severity COVID-19 using administrative claims data.

**Methods:**

A retrospective cohort study was performed using administrative claims data called Diagnosis Procedure Combination (DPC) data from advanced treatment hospital in Japan to explore potential risk factors for CAPA using multivariable regression models. In addition, the contribution of CAPA to mortality in COVID-19 patients was examined.

**Results:**

Data for 154,731 COVID-19 patients were included from DPC data. Of these, 33,136 were severe or critical COVID-19 and 14,720 were critical COVID-19. The incidence of CAPA were 0.4–1.3 % in severe or critical COVID-19, and 0.5–2.7 % in critical COVID-19. The risk factors for CAPA were steroids (hazard ratio [HR] = 4.753), immunosuppressants (HR = 2.592), intensive care unit admission (HR = 1.677), blood transfusion (HR = 6.206), and dialysis (HR = 2.185). The adjusted mortality HR for CAPA was 2.367 in severe or critical COVID-19, and 1.955 in critical COVID-19.

**Conclusion:**

The incidence of CAPA in our study was lower than previously reported. It suggests the possibility that appropriate screening test is not being conducted in Japan due to insufficient attention to CAPA. Because CAPA is a significant contributor to mortality in COVID-19 patients, appropriate screening is required in at-risk patients.

**Disclosures:**

**Hiroshi Kakeya, MD, PhD**, Asahi Kasei Pharma Corporation: Honoraria|Merck Sharp & Dohme: Honoraria|Sumitomo Pharma Co., Ltd.: Honoraria

